# Synthetic Aggregates and Bituminous Materials Based on Industrial Waste

**DOI:** 10.3390/ma17236002

**Published:** 2024-12-07

**Authors:** Alexandrina Nan, Cristina Dima, Marinela Ghita, Iolanda-Veronica Ganea, Teodora Radu, Alexander Bunge

**Affiliations:** 1 National Institute for Research and Development of Isotopic and Molecular Technologies, 67-103 Donat Str., 400293 Cluj-Napoca, Romania; alexandrina.nan@itim-cj.ro (A.N.); iolanda.ganea@itim-cj.ro (I.-V.G.); 2 Research Institute for Construction Equipment and Technology, 266 Pantelimon Road, 021652 Bucuresti, Romania; cristina.dima@icecon.ro (C.D.); marinela.ghita@icecon.ro (M.G.); 3 Faculty of Environmental Science and Engineering, “Babeș-Bolyai” University, 400294 Cluj-Napoca, Romania

**Keywords:** industrial waste, circular economy, stone dust, bituminous mastic

## Abstract

The transition to a circular economy requires new materials and products with new production designs, technologies, and processes. In order to create new materials with physico-chemical qualities suitable for application in the building materials engineering sector, stone dust and polymer waste—two environmentally hazardous industrial wastes—were combined in this study. The materials obtained were evaluated based on an analysis performed using the Micro-Deval test. The results obtained showed a Micro-Deval coefficient value of 7.7%, indicating that these artificial aggregates can replace the natural aggregates used in road construction. Additionally, it was shown that the stone dust used could be applied as a sorbent for dyes without later leaching this dye from the final synthetic stones. Another category of materials that meets the principles of the circular economy and was developed in this study is bituminous mastic, which is currently used for the hot sealing of joints in road infrastructure. For this purpose, a composite material was developed using stone dust and cooking oil to replace the filler, a non-regenerable source used for obtaining bituminous mixtures. Specific standard methods were used to assess the degree to which the new materials approach the behavior of commercially available products.

## 1. Introduction

More and more countries are increasingly interested in transitioning to a more resource-efficient and circular economy, which can contribute to long-term environmental goals and lead to job creation and economic growth [1,2,3,4,5]. It is worth mentioning that in the construction industry, an important opportunity for the circular economy is reusing industrial construction waste to significantly reduce the need for virgin building materials.

This study aimed to develop two classes of innovative materials, each involving a combination of two industrial wastes that are hazardous to health and could be used to obtain new building materials according to the principles of the circular economy. The first category of new materials with potential use in the building materials industry was obtained by combining stone dust with plastic waste. It is known that stone dust is a by-product resulting from aggregate processing for road construction, and it has yet to be used in construction or other fields so far and is, therefore, currently stored on nearby land. Some research has been dedicated to making use of stone dust produced in this manner, for example, as a fertilizer [6], sorbent for pollutants [7], or additive to concrete [8]. Plastics are currently replacing other materials such as wood or metals to a large extent due to their multiple advantages, such as the possibility of easily shaping them into different forms, their stability upon exposure to environmental conditions, their resistance to bacteria, and their light weight. Consequently, a considerable amount of waste is generated from the utilization of plastics, since a significant percentage of them is utilized for short-term applications. Much of this waste is disposed of into the environment, creating severe long-term pollution problems due to its very low biodegradability and presence in large quantities. The most common plastic wastes among the several types of plastics are polyethylene and polyethylene terephthalate. A promising sector where plastic waste can be efficiently used for various applications is the construction industry, mainly because it is the largest industry in terms of the amount of raw materials used and is, thus, the most important consumer of raw materials. In the construction industry, plastic waste can be used to produce synthetic aggregates for cementitious and asphalt mixtures, fillers, insulating materials, etc. [9]. It is worth mentioning that to our knowledge, contrary to its great potential for application in this field, its use and development are still very limited. Even though plastic waste is continuously accumulating in large quantities, currently, in the building materials industry, it is still in the development and testing stage to a relatively large extent. A relatively small number of studies have included the addition of hard-to-recycle waste plastics shredded into small pieces as an additional component to concrete aggregates in quantities that do not have a negative impact on the mechanical properties of the concrete obtained. Other studies have explored the possibility of using plastic waste as a fiber or binder while replacing aggregates to produce concrete [9], or as an additive for improving bituminous mixes and asphalt [10,11,12]. Many studies have concentrated on finding applications for this type of waste for soil stabilization or reinforcement [13,14]; as a thermal and water insulation material; as a complete alternative to traditional building materials such as bricks, plasters, paving blocks, and permeable pavements; and as an alternative to wood or plastic tiles [15,16,17,18].

Thus, as shown by earlier studies, by adopting the EU policy objective of reducing waste, plastic waste can be recycled and turned into environmentally friendly and accessible new building materials, offering several significant environmental and economic advantages and aligning with sustainable development goals [19,20].

The second class of new materials addressed in this study includes a composite based on residual oil and stone dust as an alternative to the filler currently used in the composition of bituminous mastic for the hot sealing of joints. This is a growing research area, and many results related to the utilization of industrial waste as a filler in bituminous materials have already been published [1,2,3,4,21,22].

The use of waste to replace aggregates, for example, in the construction industry, would firstly extend the natural life of quarries and secondly prevent the contamination of agricultural land areas. In addition to these mentioned advantages, the production of a new product based on industrial waste for the civil engineering sector could generate multiple economic and environmental benefits by mitigating their environmental impact and reducing the consumption of materials from non-renewable sources.

The use of plastic waste in the civil construction industry has increased in the last few decades in most cases, and different types of plastic waste have been utilized in concrete or mortars to substitute natural aggregates, either as fine or coarse aggregates. Many studies have been conducted to observe the physical and mechanical behavior of construction materials with plastic waste used as a fine or coarse aggregate replacement [23]. The advantages observed in the construction materials containing plastic waste were the following: a lower unit weight and density compared to materials without plastic replacement and a decrease in water absorption due to a reduction in material capillary voids, since the plastic has a lower water affinity. However, a detailed feasibility study and lifecycle evaluation should be performed to study and provide a more detailed understanding of the environmental effects.

Hot mix asphalts are the materials most used for road pavements in the construction industry. They are composed of aggregates, bitumen, filler, and different types of additives for both processing and performance purposes. Bitumen and filler form together the so-called mastic, which has a crucial role in the bituminous-based material’s performance because it has to bear both traffic loads and climatic changes under exposure to real environmental applications. Besides environmental issues, inflation in the cost of materials from non-regenerable sources and the gradually declining amount of natural resources have forced decision makers to utilize waste/secondary materials as replacements for conventional construction materials. Stone dust plays a double role in the bituminous mastic: it fills the interstices between aggregates and thus provides improved mechanical strength and impermeability, thus modifying the viscosity of the obtained material. Therefore, the physico-chemical characterization of the stone dust is required before using it as a substitute for commercially available filler.

Thus, to achieve what we set out to do in this study, several materials were prepared as synthetic aggregates using stone dust and four different types of plastic waste. Validation of each of the obtained materials by standardized tests was carried out to assess the suitability of such waste materials for synthetic aggregate production. The stone dust was also tested for its sorption capacity for several synthetic dyes, and aggregates were produced from the spent sorbent (i.e., stone dust with dye adsorbed on it). The leaching of dye from the final synthetic rocks was also tested.

Secondly, a composite material was developed using stone dust and waste cooking oil to replace filler, a non-regenerable component used for obtaining bituminous mixtures. The waste-based composite will replace portions of the filler’s initial content to maintain the bituminous mastic’s physical and mechanical properties, while minimizing the use of natural resources. The materials will be analyzed using specific standard methods to determine their characteristics and to assess the degree to which the new material replicates the behavior of the commercially available product.

It is worth mentioning that the lifecycle assessment (LCA) of the materials developed in this study highlights their potential environmental benefits and challenges. We identified the following details regarding the LCA: the use of industrial waste materials, including stone dust, plastic waste, and used cooking oil, significantly reduces the reliance on non-renewable resources and aligns with circular economy principles as compared to conventional materials that either require quarrying, which has a high environmental cost, or which are derived from traditional resources. Stone dust, a by-product of aggregate production, is utilized both as a sorbent for dyes and as a component in synthetic aggregates, minimizing its environmental disposal impact. Similarly, including plastic waste and cooking oil diverts these materials from landfills, mitigating pollution and waste accumulation. These substitutions provide clear advantages over conventional materials such as virgin aggregates and fillers, which require resource-intensive extraction and processing. However, we are aware that these materials’ production processes are energy-intensive, particularly the heating stages required for synthesizing them. However, recycling these aggregate wastes should reduce the huge extractive energy consumption used in the building sector. Moreover, these steps can contribute to greenhouse gas emissions and may offset some environmental gains unless further optimized. Additionally, the potential for microplastic release during the lifecycle of synthetic aggregates requires further investigation.

Despite these challenges, the materials demonstrate strong potential for reducing the environmental footprint of construction practices by addressing waste reuse and resource efficiency. This way, we can help significantly diminish the use of traditional raw materials, aligning with circular economy goals, as well as mitigating plastic waste pollution and reducing landfill loads.

## 2. Materials and Methods

### 2.1. Materials

**Figure 1:**
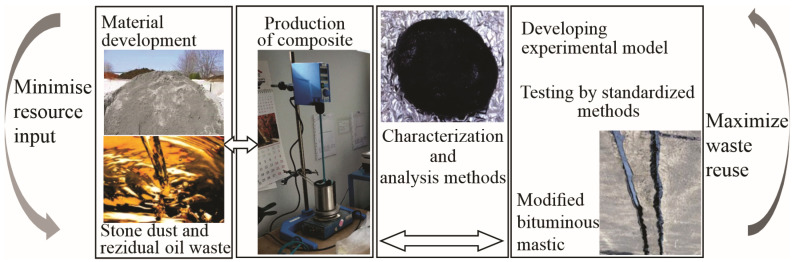
Schematic representation of the steps necessary to carry out the proposed study. In the first stage, the bituminous materials will be prepared. Characterization of the materials obtained will be performed, and based on the results achieved, the preparation method will be optimized. Evaluation of mechanical performance and durability by specific testing protocols will be performed. The arrows between stage blocks indicate the feedback loop for material preparation and testing. After their evaluations, the most promising materials for the targeted applications will be selected.

The stone dust waste used in this study comes from the northwestern part of the Huedin region in Romania. Polymer-modified bitumen type PMB 45/80-65 from the provider Mol-Hungary was used as a binder. Waste from cooking oil was collected after its use as frying oil in the food industry. The waste tire rubber powder used in this study has a fine powder appearance with a particle size < 1 mm and was obtained from the supplier Iterchimica. According to the provider, it is a black powder with a 120−150 °C softening point and an uncompressed density higher than 0.3 g/cm^3^. Crystal violet from Loba Chemie Fischamend; methylene blue from Merck, Germany; fast green FCF from Alfa Aesar (Thermo Fisher, Kandel, Germany); and Congo red from Reactivul București (Bucharest, Romania) were used for adsorption tests. A schematic representation of the steps necessary to carry out the proposed study is shown in Figure 1.

### 2.2. Preparation of Synthetic Aggregates

For the preparation of samples based on stone and plastic dust, four types of polymers were used: polyethylene terephthalate (PET), polyethylene (PE), latex, and polypropylene (PP) plastic; the description of the samples is found in Table 1. The plastic was cut into pieces of a small width—approximately 5 mm—mixed with well-determined quantities of stone dust, and placed in the preheated oven (as indicated in Table 1) for 18 h to prepare the samples. Then, they were taken out of the oven, homogenized, and left to cool to room temperature.

### 2.3. Preparation of Bituminous Materials

For the preparation of waste-modified bituminous mastic, composite samples were prepared from stone dust and oil in different ratios to be introduced into the mastic matrix. The bitumen preheated in the oven to the flow temperature was placed in a metal pot with a capacity of 6 L, positioned on an electric hob at a temperature of 160 °C. A mixer in the form of a metal coil was then inserted into the vessel, with the upper end fixed in a rotary motor positioned above the vessel with the help of a stand. After adding the solid part to the bituminous material, it was continuously mixed for 30 min at a constant temperature and speed, then removed from the pot and left to cool to room temperature. The exact composition can be found in Table 2.

### 2.4. Methods

The characteristic parameters of the stone dust, such as the surface area, pore volume, and pore size, were determined using a constant temperature adsorption/desorption technique of nitrogen molecules on the solid surface. The specific surface area determined on the analyzed sample was approximately 1.5 m^2^/g of the variation of the adsorbed volume with pressure. The average pore volume determined was 0.006 cm^3^/g [22].

The Micro-Deval Wear Resistance method determined the prepared composites’ Micro-Deval coefficient in relation to natural aggregates of similar dimensions [24]. This method measures the abrasion resistance and durability of mineral aggregates in the presence of water and an abrasive charge, and has been accepted as a European Union standard test (EN 1097-1, [25]). Since some wet materials vary their hardness compared to those in a dry state, using water in this test evaluates the decrease in resistance to degradation.

Samples of waste-modified bituminous mastic asphalt were tested by determining density and cone penetration at 25 °C according to the standard SR EN 13880-2:2004 [26]. After preparation and conditioning of the test pieces, the cone penetration depth was determined at a temperature of 25.0 ± 0.3 °C, with an applied load of 150.0 ± 0.1 g and a load application time of 5.0 ± 0.1 s. The softening point, which is the temperature at which the material, under standard test conditions, reaches the specified consistency, was determined according to the current standard SR EN 1427:2002 [27].

Using the Hot Disk TPS 2500S (Hot Disk AB, Kagaku, Sweden), thermal conductivity was measured using the transient plane source (TPS) method. This method implies that voltage variations are measured at the TPS sensor during the experiment, while a constant pulse (applied during the measurement) slightly increases its temperature. The sensor is mounted between two identical cylindrical-shaped samples to ensure good thermal contact. The equipment also considers the possibility of analyzing the diffusivity and specific heat of materials.

Compressive measurements were performed on selected samples of commercial mastic and waste-added modified mastic in the temperature range of −30 to 70 °C using a Dynamic Mechanical Analyzer (DMA) 7100 supplied by Hitachi Ltd., Tokyo, Japan, to analyze the behavior of the samples by mechanical testing. These tests were performed on samples prepared to have cylindrical shapes with dimensions of 15 mm × 13.5 mm.

The measurement parameters used were: temperature sweep mode (heating rate 1 °C/min) applied with a frequency of 8 Hz, compressive force of 100 mN, compressive force gain of 1.5, and force amplitude of 100 mN. The storage modulus (G″) and loss modulus (G″) were recorded as a function of temperature.

Fatigue testing of waste-modified bituminous mastic materials evaluated the elastic resilience, a specific characteristic of hot-applied joint sealants.

In order to carry out this experiment, the material was hot poured between two concrete slabs with dimensions of 200 × 50 × 30 mm (L × W × H), positioned at a distance of 10 mm from each other. The final dimensions of the mastic molded in the joint are 50 × 10 × 30 mm. The samples were conditioned at 23 °C and 55 ± 5% RH for 7 days. During the test, the samples were subjected to 1000 oscillation cycles at a velocity of 10 ± 2 mm/h, at an amplitude between 0 and 2 mm and a temperature of 23 °C.

### 2.5. Adsorption/Leaching Tests

Adsorption tests were carried out as follows: Calibration curves were created using UVVIS spectrophotometry for defined concentrations of aqueous solutions of methylene blue (absorbance determined for 665 nm), crystal violet (590 nm), fast green FCF (625 nm), and Congo Red (500 nm). For determination of removal efficiency and sorption capacity, ca. 1 g/ca., 100 mg of stone dust was accurately weighed, and an aqueous solution of the respective dye (methylene blue, crystal violet, fast green FCF or Congo Red, 250 mg/L, 20 mL) was added. After stirring for 17 h, the suspension was filtered, and the dye concentration in the filtrate was determined by UV–VIS spectroscopy.

For the leaching test, a piece of synthetic stone (3.6 g, prepared from stone dust on which crystal violet was adsorbed) was left to stand in water (30 mL). After 4, 8, 12, and 16 days, aliquots were taken and analyzed for dye content by UV–VIS spectroscopy.

## 3. Results

**Figure 2:**
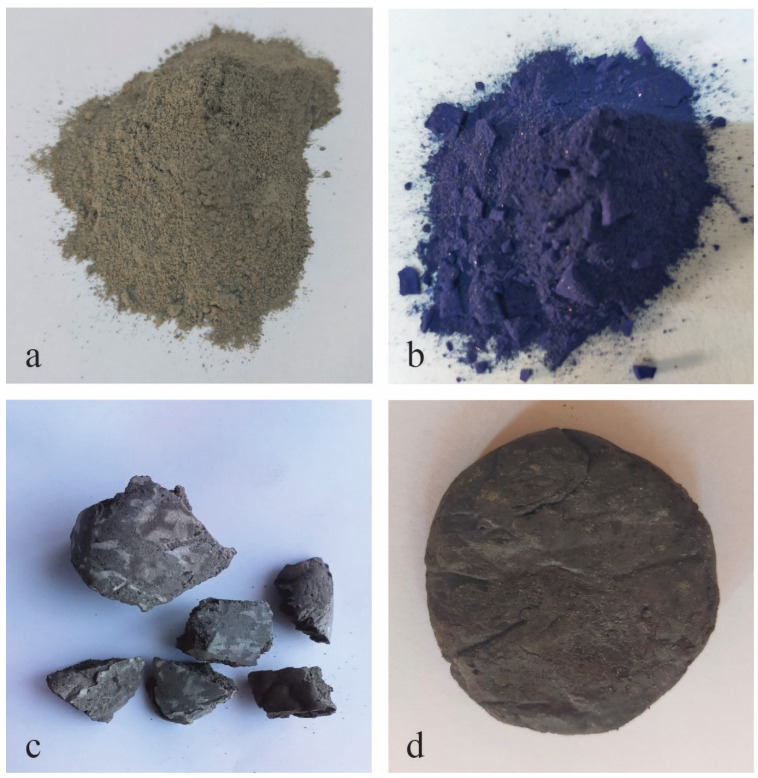
Stone dust (**a**), stone dust after CV adsorption (**b**), example of the S2 synthetic aggregates (**c**), example of synthetic aggregates with CV-embedded stone dust (**d**).

Usually, stone dust waste (Figure 2a) consists of particles in the size range of 0.01–1 µm [22]. Therefore, it could be easily mixed with shredded plastic waste. In addition to its unit weight and ductility, plastic waste is a feasible component for producing lightweight composites that is strong due to its stiffness, shear response, and load-bearing capacity.

Among the composites prepared under the conditions presented in Table 2, sample S2 presents the best premises in terms of the targeted application. Figure 2c shows the obtained sample, which is solid and has a rough appearance similar to that of stones. This result can be understood because, at a temperature of 180 °C, the waste plastic melted and interacted uniformly throughout the whole mass with the fine dust particles. The results obtained for the waste plastic used to prepare samples S1 and S3 suggest that the melting of the plastic material did not occur at 200 °C, which led to the failure of the material formation. However, further tests at higher temperatures are needed for a better understanding of the thermal behavior of these compositions. As an observation in the case of sample S4, there was a partial melting of the latex in the presence of dust particles, but the obtained composite material shows a soft consistency and increased elasticity.

Table 3 presents the results obtained for the Micro-Deval coefficient for the investigated compositions in relation to similar-sized natural aggregates, type 10–14, used as a reference material (RM).

The Micro-Deval Test has earned recognition and popularity recently as an economical and accurate method for aggregate abrasion testing. This method provides a measure of the toughness, abrasion resistance, and durability of different aggregates as they are ground with an abrasive charge in the presence of water. In the experimental setup, aggregates that interact with each other are exposed to steel balls and water in a steel container that rotates during the experiment. After the end of the test time, the Micro-Deval coefficient was calculated, which describes the weight loss resulting from exposure to polishing. A higher coefficient corresponds to a lower wearing resistance of the analyzed aggregates. The Micro-Deval test carried out on the obtained S1–S4 materials (Table 3) indicated a Micro-Deval coefficient with a value of 7.7% for S2, which indicates that these particular artificial aggregates can replace the natural aggregates currently used in road construction.

There is a continuously increasing interest in establishing low-cost techniques for efficiently removing organic/inorganic pollutants through adsorption techniques, using waste materials from industrial, domestic, and agricultural activities. Andesite stone dust has been previously used as a sorbent for cationic dyes such as methylene blue [7], as well as cations such as nickel [28] or mercury [29]. It has a high potential to be used as a sorbent for water purification because of its availability in large quantities and low (possibly even below zero) price. Adsorption is one of the water purification methods that the most research has been carried out on [30], due to its high versatility. However, it also has some drawbacks. One of these drawbacks is that adsorption generates large amounts of waste in the form of spent sorbent. The spent sorbents can often be regenerated afterward [31]. However, alternative solutions exist [32]. In the search for new and cheap possibilities for the adsorption and reuse of spent sorbent, the adsorption capacity of stone dust waste was analyzed by immersing it in solutions prepared using four different dyes: methylene blue, crystal violet, fast green FCF, and Congo Red. These represent different types of dyes, both regarding their use and structural motifs, as well as their electronic properties (cationic/anionic) [33]. In order to demonstrate a way to reuse the sorbent after adsorption, stone dust with adsorbed crystal violet was subjected to the same conditions as described above to obtain synthetic stones using polypropylene waste, and the leaching of dye in water was tested.

The determined removal efficiencies and sorption capacities for the four dyes are listed in Table 4. It should be mentioned that these values were determined by leaving the sorption process for more than an order of magnitude longer than what was determined for methylene blue to be the time after which almost no change had occurred (17 h) [7]. As such, the values determined here might differ slightly from values determined using multiple different sorption experiments, fitting, and extrapolation, but the difference should be small enough to be negligible. Several findings in these experiments seem noteworthy: First, except for fast green FCF (which has a quite low sorption capacity of 0.5 mg/g), the sorption capacities of the other three dyes were in a comparable range. Overall, the sorption capacities showed a similar trend as with other sorbents [33], in that cationic dyes get adsorbed better than anionic ones (fast green FCF), which shows that the surface likely also exhibits anionic groups. The absolute values are lower than in [33], likely due to the igneous andesite dust exhibiting no pores and, thus, having a comparatively low specific surface. Interestingly, despite showing a lower specific surface of 1.5 m^2^/g than a similar material researched before [7], the sorption capacity for methylene blue is far higher than described in this source. Differences in the composition of stone dust may play a role here. However, another explanation could be the use of different pretreatments, since the source of rock dust, in this case, is from a road construction company. For dye amounts lower than the sorption capacity, almost all dye could be adsorbed from the water (Table 1), with removal efficiencies reaching more than 98%. This shows that andesite stone dust is an excellent means of removing several synthetic dyes from polluted water.

In order to reuse the spent sorbent, a larger quantity of stone dust was subjected to a solution of crystal violet, the dye showing the highest sorption capacity of the four tested. After drying, it was possible to notice the adsorbed dye easily, as the stone dust had changed color to purple (Figure 2b). Synthetic stones were then synthesized using this modified stone dust and polypropylene waste, using mass ratios for the S2 composition. The stones that were thus obtained were subjected to leaching by immersing them in water for several days. An exemplification of the obtained material is shown in Figure 2d. The morphological aspect and hardness are similar to the initially obtained S2 sample, but its color is different due to the crystal violet content in the composition. Aliquots were taken every four days. However, for the time observed (16 days), no crystal violet could be found in the supernatant (determined specific desorption < 0.1 µg/g, and thus, below the limit of detection). This proves that synthetic stones made in this way cannot only turn a waste product (andesite stone dust) into a valuable product (aggregate), but also can intermittently be used as sorbent, the dye afterward not leaching from the prepared synthetic stones.

The thermal conductivity, diffusivity, and specific heat were determined for synthetic stones and compared to the data on commercial filler and stone dust. The results obtained are shown in Table 5. As indicated in Table 5, synthetic stone has a lower conductivity and specific heat, but higher diffusivity than the other compounds studied, which can be beneficial from the point of view of the material’s thermal stability when used in the targeted applications [22]. Moreover, the low thermal conductivity value shows that the material can work as a highly energy-efficient construction material.

Regarding the second class of materials based on industrial waste, batches of homogeneous samples in hundreds of grams were initially obtained with the composition indicated in Table 2. For the obtained mastic, the main characteristics presented in Table 5 were determined by specific methods, as shown previously [22]. Thus, these compositions are optimized based on our previous research that identified the optimal proportions for achieving desirable mechanical and thermal properties.

It is worth mentioning that for such materials, the following performance is specified as the essential requirements for use in targeted applications: homogeneous composition, softening point > 85 °C, penetration at 25 °C in the range of 40–100, and density at 25 °C [34]. Table 6 shows that samples M4 and M6 fulfilled the requirements because they have the largest values for the softening point, at 108.9 and 105.3 °C, respectively.

**Figure 3:**
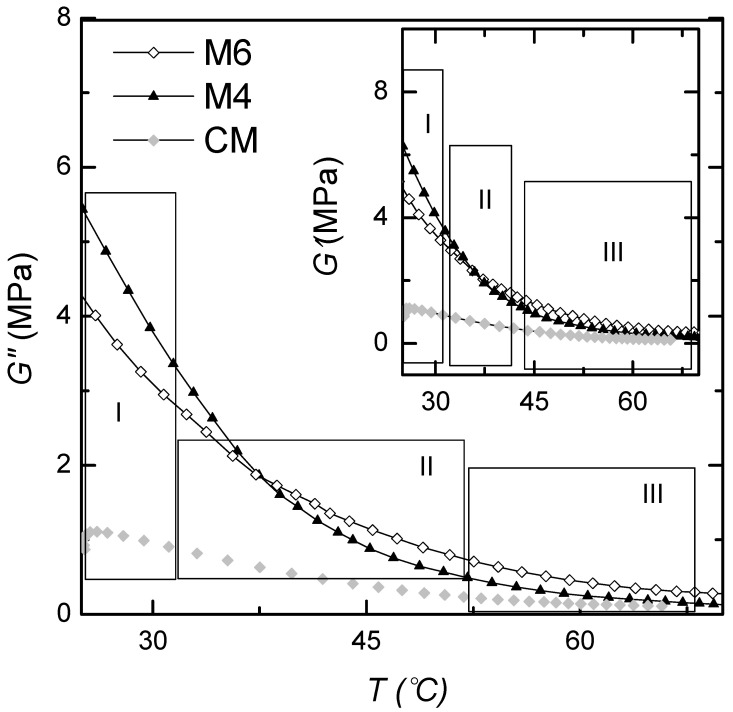
Direct comparison of dissipation module *G*″ and storage module *G*′ for M4 and M6 and commercial mastic (CM).

Figure 3 shows the variation of *G*″ and *G*′ as a function of temperature for samples M4, M6, and commercial mastic (CM). *G*″ can be defined as a viscous response of the material or as the susceptibility to dissipate the applied energy, and as can be seen, had a similar behavior for all the materials studied. *G*″ decreases slightly with increasing temperatures, up to approximately 30 °C (Zone I). This is followed by a steep decrease to approximately 50 °C (Zone II). In the analyzed temperature range, *G*″ has the maximum value for sample M4. In contrast, for CM, it has a minimal value. The observed difference could be due to the differences in the composition of the three samples. Table 2 shows that sample M6 contains a more significant amount of stone dust, 150 g, while sample M4 contains 102 g. However, *G*″ is expected to indeed be larger for materials with less bitumen because it is directly affected by the “internal friction” of stone dust particles. With temperature increases above approximately 30 °C (Zone III), *G*″ has a very low variation, similar for all the samples.

The inset of Figure 3 shows the temperature dependency of *G*′, known as the dynamic mode. It is a measure of the elastic energy stored by a material, and as can be seen, it has a similar behavior concerning temperature for all the investigated samples. However, three distinct regions can be identified: the first region (I) occurs at temperatures T < 30 °C, where the storage module shows a drastic decrease with increasing temperatures, which suggests the fact that the material presents itself as a very hard solid (vitreous state); the second region, II, called the glass transition since the magnitude of the storage module shows a slight decrease with temperature from −30 °C to approximatively 50 °C; the third region, III, is in the temperature range of 45–70 °C, in which *G*′ behaves differently as the material changes from rigid to soft rubber. The curves obtained level out, with *G*″ decreasing very slowly with increasing temperatures over the entire measuring range, up to 70 °C. The results suggest that these materials present good hardness and high compressive strength in the targeted temperature range.

Based on the results obtained, larger quantities of M4 and M6 samples were prepared to verify that they maintained their properties after upscaling. Table 7 shows the composition of the two selected samples after scaling up to 2400 g. The newly prepared samples have been named M4-S and M6-S.

**Figure 4:**
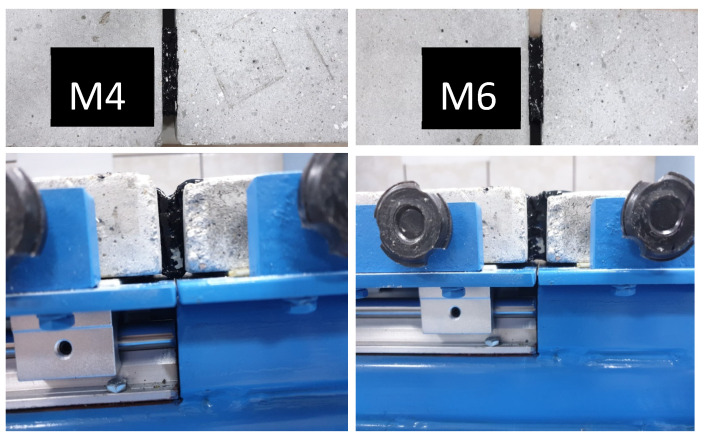
Exemplification for testing selected compositions in exposure conditions similar to real-life ones: variations in temperature, pressure, and mechanical vibrations.

**Figure 5:**
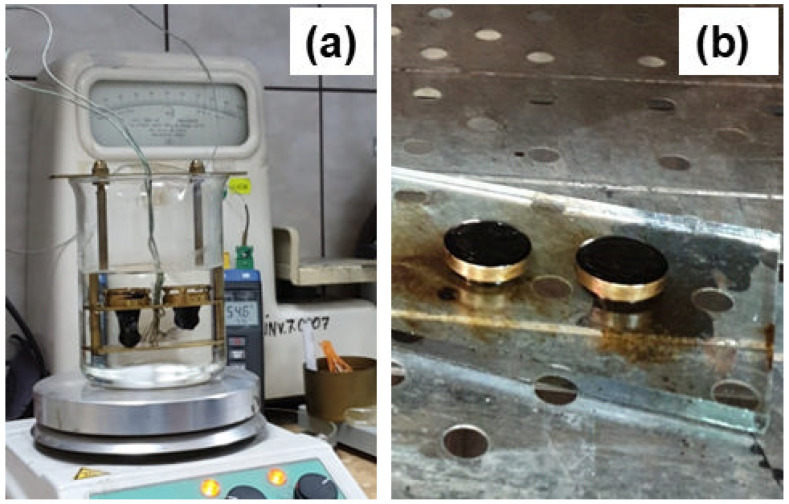
Determination of the softening point using a ball (**a**) and ring (**b**), according to SR EN 1427:2002. The determined soaking point is the temperature at which the bituminous material with added waste under standard test conditions reaches the specified consistency.

It is known that bituminous materials from road structures are exposed to short-term loading during vehicular traffic. This loading causes a loss of rigidity in the bituminous material and can lead to failure by accumulation during long-term exposure. Thus, the resulting fatigue degradation is of great importance in road infrastructure and must be understood correctly to ensure adequate compositions for the targeted applications. Our experiments show that after performing the fatigue test, sample M4 went into the flow after 600 cycles, while sample M6 went into the flow after 850 test cycles (Figure 4). This different behavior of the two compositions, that has been observed comparatively, may be understood by their softening point values. The experimental setup for this type of measurement is shown in Figure 5. Sample M4-S has a softening point lower than that determined for the M6-S, as shown in Table 8. The difference observed may appear because, unlike M6-S, for which polymer-modified bitumen was used for the preparation, M4-S was prepared using classic bitumen. Thus, unmodified bitumen, although more cost-efficient, is less suitable than polymer-modified bitumen for real application in road infrastructure. However, for the M6-S, the softening point is still in the range of values accepted by the standards currently used for this class of materials [34]. Another factor to consider when comparing the results is the quantity of stone dust used. An increase in softening point is generally related to an increase in stiffness, which in our case, can be attributed to the fact that M6-S has a higher number of particles in its composition than M4-S relative to bitumen quantity, as seen in Table 7.

A recent related study on the behavior of asphalt mastics containing five different materials as a filler (natural limestone, hydrated lime, Portland cement, Linz–Donawitz steel slag, and blast furnace slag) was reported [35]. Results obtained mainly on the penetration, softening point, viscosity, multiple stress creep, and recovery tests showed that the bituminous mastic properties depend on the composition and test types performed. It has been shown that an increase in the softening point is directly related to an increase in stiffness, mainly due to the physicochemical interactions of the active fillers with the asphalt, but also due to the adsorption process of some of the lighter components of the bitumen composition in the pores of the mineral filer [36].

By comparing the results obtained for the M4-S and M6-S samples with their equivalents, obtained in quantities of hundreds of grams, their softening point is higher compared to scaled-up samples.

## 4. Conclusions

Based on the results presented in this study, we demonstrate that two classes of materials can be developed using three different types of industrial hazardous waste: stone dust, plastic of polypropylene type, and cooking oil. Stone dust and polypropylene plastic generate a material that possesses a mechanical hardness compared to the natural aggregates. The results obtained in the present study show that by using two industrial wastes, real progress can be made in protecting the environment by obtaining synthetic aggregates that can at least partially replace natural aggregates, which are increasingly expensive resources and come from non-renewable sources. Thus, the economic and environmental advantage, together with the simplicity of the preparation method of these composite materials, is an important motivation for their use in civil construction. The value generated by this is even higher, considering that before using stone dust to prepare synthetic rocks, it can be used as a sorbent for water decontamination without affecting the final product.

In the case of bituminous mastic materials, through specific standard tests, it was observed that using the mass ratios presented in Table 7, bituminous mastic materials that meet the specific standards in the field of construction materials currently used in road infrastructure can be produced. Based on the results obtained through upscaling and testing the compositions at the laboratory level, a technological maturity has been reached that allows us to move on to tests under real-life exposure conditions with interested beneficiaries.

## Figures and Tables

**Table 1:** Samples obtained from two industrial wastes: stone dust and plastic.

Sample	Stone Dust (g)	Plastic Waste	Type of Plastic Waste	Temperature (°C)	Time (h)
S1	10	2.5	PE	180	18
S2	30	10	PP	180	18
S3	30	8	PET	200	18
S4	55	13	Latex	180	18

**Table 2:** Composition of the bituminous mastic samples.

Sample	Bitumen Quantity (g)	Filler Quantity (g)	Stone Dust Quantity (g)	Residual Oil Quantity (g)	Rubber Powder Quantity (g)	Total Mass (g)
M1	459	102	0	0	39	600
M2	459	51	51	0	39	600
M3	459	0	102	0	39	600
M4	459 *	0	102	0	39	600
M5	438.6	0	102	20.4	39	600
M6	381	0	150	30	39	600
M7	429	0	102	30	39	600
M8	459	0	102	0	39	600
M9	457	0	102	20	20	600

* marks that classic bitumen without polymer in the composition was used for this sample.

**Table 3:** Micro-Deval parameters used to analyze the prepared materials.

Materials (g)	Mass Percentage (%)	Micro-Deval Coefficient (%)	Standard Conditions (%)	Observations
RM = 500	500 g = 100%	7.6%	Max 15%	
RM = 450 S1 = 50	450 g = 90% 50 g = 10%	14.9%	Max 15%	The material was destroyed
RM = 450 S2 = 50	450 g = 90% 50 g = 10%	16.4%	Max 15%	The material remained intact
RM = 450 S3 = 50	450 g = 90% 50 g = 10%	16.6%	Max 15%	The material was destroyed
RM = 450 S4 = 35	465 g = 93% 35 g = 7%	7.7%	Max 15%	The material was destroyed

**Table 4:** Removal efficiency and adsorption capacity of stone dust determined for the four dyes.

Adsorbed Dye	Removal Efficiency (%) (5 mg Dye/g Stone Dust Initial Amount)	Adsorption Capacity (mg/g) (50 mg Dye/g Stone Dust Initial Amount)
Methylene blue	>99.9	30.2
Crystal violet	99.9	44.4
Fast green FCF	10.8	0.50 *
Congo Red	98.9	41.7

* = 5 mg dye/g stone dust initial amount.

**Table 5:** Thermal properties of limestone, commercial filler, and synthetic aggregates.

Filler	Thermal Conductivity (W/mK)	Thermal Diffusivity (mm^2^/s)	Specific Heat (J/gK)
Limestone	2.8780	1.797	0.626
Stone dust	0.4399	0.2096	2.100
Synthetic stone	0.113	2.342	0.048

**Table 6:** Specific parameters (softening point, density, penetration) determined according to standards for the obtained materials.

Sample	Density (g/cm^3^)	Softening Point (°C)	Penetration 0.1 mm
M1	1.109	63.4	49
M2	1.106	68.8	40
M3	1.072	63.2	51
M4	1.068	108.9	51
M5	1.033	78.3	45
M6	1.151	105.3	50
M7	1.046	77.4	52
M8	1.250	48.6	56
M9	1.070	55.6	56

**Table 7:** The composition of the samples for which upscaling was performed.

Sample	Bitumen Quantity (g)	Filler Quantity (g)	Stone Dust Quantity (g)	Residual Oil Quantity (g)	Powder Quantity (g)	Total Mass (g)
M4-S	1836	0	408	0	156	2400
M6-S	1524	0	600	120	156	2400

**Table 8:** Specific parameters were determined for selected M4 and M6 samples, according to standards.

Specific Parameter Determined	Test Method	Results-M4-S	Results-M6-S
Density, g/cm^3^	SR EN 13880 [26]	1.067	1.167
Softening point: Ring and ball method	SR EN 1427 [27]	55	89.2
Cone Penetration at 25 °C, 0.1 mm	SR EN 13880 [26]	28	25
Cone penetration after aging 168 h, 0.1 mm	SR EN 13880 [26]	25	23
Fatigue test at 23 °C, 600 cycles with oscillation 0 ÷ 2 mm	-	The sample melted and flowed	No visual changes

## Data Availability

The original contributions presented in the study are included in the article, further inquiries can be directed to the corresponding authors.
